# Dynamic Changes of Generic Quality of Life after Different Treatments for Localized Prostate Cancer

**DOI:** 10.3390/jcm10010158

**Published:** 2021-01-05

**Authors:** Yao-Lin Kao, Chien-Hui Ou, Sheng-Hsiang Lin, Sheng-Mao Chang, Jung-Der Wang, Yuh-Shyan Tsai

**Affiliations:** 1 Department of Urology, National Cheng Kung University Hospital, College of Medicine, National Cheng Kung University, Tainan 704, Taiwan; pleasewaitforg@hotmail.com (Y.-L.K.); donou1969@yahoo.com.tw (C.-H.O.); 2 Institute of Clinical Medicine, College of Medicine, National Cheng Kung University, Tainan 704, Taiwan; shlin922@mail.ncku.edu.tw; 3 Department of Public Health, College of Medicine, National Cheng Kung University, Tainan 704, Taiwan; jdwang121@gmail.com; 4 Biostatistics Consulting Center, National Cheng Kung University Hospital, College of Medicine, National Cheng Kung University, Tainan 704, Taiwan; 5 Department of Statistics, National Cheng Kung University, Tainan 701, Taiwan; smchang@mail.ncku.edu.tw

**Keywords:** prostate cancer, quality of life, prostatectomy, radiotherapy, active surveillance

## Abstract

Generic quality of life (QoL) is an important issue in decision making related to the primary treatment of localized prostate cancer (PC). This study assessed the dynamic changes of QoL in patients with localized PC under different treatment modalities. From 2013 to 2018, we prospectively assessed QoL scores in patients with localized PC under unitary treatment using the World Health Organization Quality of Life (WHOQOL) BREF version. The trajectories of the QoL scores after different treatments were estimated using a kernel-smoothing method. Dynamic changes in the major determinants were analyzed using a mixed effects model. The clinical features of the participants in our institute were compared with PC patients in Taiwan’s cancer registry. A total of 196 patients were enrolled with 491 repeated assessments. The participants shared similar clinical characteristics with the PC patients in Taiwan as a whole. Patients with lower household incomes showed statistically significant lower scores on all four domains and related facets, while PC survivors with comorbidities of anxiety and/or diabetes appeared to be affected on the physical domain and related facets. After controlling for these determinants, patients under active surveillance or observation demonstrated significantly higher QoL scores in the physical and social domains, as well as several facets belonging to these domains, in mixed models compared with patients undergoing radical prostatectomy or radiotherapy within the first year. The generic QoL scores were higher within the first year in patients receiving active surveillance or observation after controlling other significant factors. The difference diminished after one year of post management. More studies are needed to corroborate our findings.

## 1. Introduction

Prostate cancer (PC) is the most common non-cutaneous male malignancy in developed countries [1]. Most patients diagnosed in the early phase as having localized PC have a five-year relative survival rate greater than 99% [2]. Current clinical guidelines recommend that patients with localized PC receive management including radical prostatectomy (RP), external beam radiotherapy (RT), or active surveillance (AS) [3]. While these management techniques have comparable cancer-specific survival rates based on long-term follow-up [4], each treatment has unique side effects for PC survivors, such as urinary incontinence in RP, bowel function in RT, and anxiety in AS [5,6]. Therefore, quality of life (QoL) is an important issue in the decision-making process for primary PC management.

The evaluation of QoL in PC patients is comprised of both generic QoL and cancer specific QoL [7]. Generic or global QoL assessment serves as an important predictor of individual health, which is a multidimensional concept encompassing physical, psychological, social, and environmental health [8], which is not limited to the presence of specific treatment-related side effects. Previous studies have often failed to identify the differences in generic QoL among different primary management options [9]. Generic QoL differences may be attenuated by subsequent treatments. In addition, variations in dynamic QoL may not be detected in analyses other than continuous curve evaluations. However, our earlier study found differences in generic QoL among PC patients with different sociodemographic conditions [10]. In this study, we aim toward assessing the differences in generic QoL in localized PC patients.

## 2. Materials and Methods

This study was commenced after the approval of the Institutional Review Board of the National Cheng Kung University Hospital, a tertiary medical center in southwest Taiwan (NCKUH; A-ER-101-219; 1 January 2013).

### 2.1. Patient Population

From January 2013 to December 2018, patients with localized PC visiting our urology outpatient department (OPD) were invited to participate in the study. They were encouraged to voluntarily complete the QoL questionnaire during the OPD visit. Repeated QoL assessments were performed with the same patients during long term OPD follow-up, at least two weeks apart, for evaluation of the dynamic changes in QoL.

Participants diagnosed with localized PC who were receiving RP, RT, or AS treatment and who had filled out the QoL questionnaire at least once were included in the final analysis. Patients under observation were also included in the AS group. According to the 2020 National Comprehensive Cancer Network (NCCN) guidelines for prostate cancer, localized PC was defined as a tumor confined to the prostate and its nearby region, without evidence of seminal vesicle invasion or distal metastasis as the stage < T3b with N0 and M0.

Patients with PSA > 100 ng/mL were excluded so as to avoid a potential high risk of occult metastasis. Patients with any history of neurogenic bladder or previous urinary tract complications were excluded because of potential confounding effects. As we were exploring the pure effects of different primary treatment options for prostate cancer, participants were censored at the time they received subsequent treatment. Education, income, and employment were retrieved at the time of the first assessment. Clinical data of prostate size at diagnosis and the D’Amico risk group were retrieved from electronic medical records. Age and comorbidities, including cardiovascular disease, diabetes, hypertension, arthritis, anxiety/insomnia/depression, and peptic ulcer, that might affect PC treatment outcomes [11] were repeatedly assessed and recorded at each QoL assessment during follow-up.

To test the representativeness of our participants, we further obtained the clinical data of patients with localized PC diagnosed in 2009 to 2016 from the Taiwan Cancer Registry (TCR), which covered nearly the entire PC diagnosis time span in our study.

### 2.2. QoL Questionnaire

The patients’ QoL was evaluated with the Taiwan version of the World Health Organization Quality of Life-BREF (WHOQOL-BREF). It is a widely used, validated, generic psychometric instrument [12] that is also sensitive to the QoL of PC patients [13]. The questionnaire has been translated into more than 40 language versions and includes questions that not only assess the presence of side effects and symptoms, but also assess the participants’ degree of satisfaction with their life, which might serve as an ideal tool for generic and cross-cultural QoL assessment [14].

WHOQOL-BREF contains two items for the overall assessment and four major domains, including physical capacity, psychological well-being, social relationships, and environment, with seven, six, four, and nine items within each domain, respectively. Each item is ranked on a five-point Likert score, where higher scores represent a better QoL. The domain score is calculated as the average of its items multiplied by four, and ranges from 4 to 20 [15].

### 2.3. Trajectories of QoL Change after Treatments

The QoL outcome trajectories at different timepoints after each PC treatment were constructed using Gaussian kernel-smoothing [16]. It builds the continuous QoL function at each timepoint via a calculated weighted average of the nearest 10% of the neighboring values [17]. A 95% confidence interval of the mean QoL function at each time point was further created using a bootstrap approach. Details of the techniques are described in our previous studies [18].

### 2.4. Statistical Analyses

The distributions of the baseline clinical and socio-demographic information among the different PC treatment groups were analyzed via either a chi-square test or Fisher’s exact test. A comparison of the clinical characteristics between our participants and those with localized PC in the TCR were analyzed with a Student’s *t*-test and a chi-square test as continuous and non-continuous variables, respectively.

A linear mixed effects model was constructed to quantify the changes in the scores for the major determinants (or fixed effects) of QoL and the random effects for repeated assessments in the same patients. Missing values were deleted in the analysis, which accounted for 2.8% of all the measurements. Analyses were conducted using Statistical Analysis System^®^ software version 9.4 (SAS Institute, Cary, NC, USA). Two-sided *p*-values below 0.05 were considered significant.

## 3. Results

A total of 196 patients with localized PC receiving standard cancer management were enrolled in the outpatient department of urology at National Cheng Kung University Hospital (NCKUH); 491 assessments were performed on these patients. The clinical and sociodemographic characteristics of the patients stratified according to PC treatment are summarized in Table 1.

There were 31,183 patients diagnosed with localized PC in the TCR. The clinical characteristics of the participants at NCKUH and the patients in the TCR are summarized in Table 2. Our participants were slightly younger compared with those in the TCR. Except for arthritis, there were no significant differences in other major comorbidities, the PC risk group, and the primary treatment modalities of our participants and the patients in the nation as a whole.

A younger age at diagnosis, better education, and incomes with a higher proportion of employment were noted in patients receiving RP compared with the other two groups. Patients with a higher risk of D’Amico risk stratification appeared more likely to receive RT, while those with a low or moderate risk received RP or AS more frequently. No statistical differences in the distributions of major comorbidities, total activities of daily living scores, initial WHOQOL-BREF domain scores, or prostate size were noted among the different groups.

### 3.1. Changes in QoL after Different Prostate Cancer Treatment

**Figure 1:**
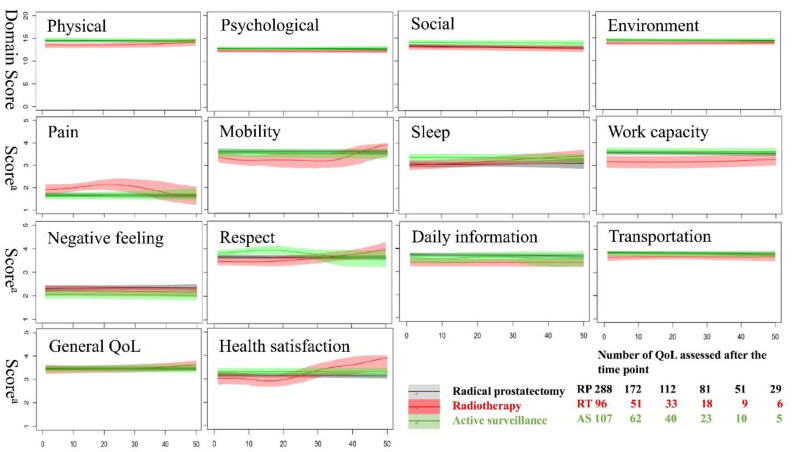
Dynamic changes in the World Health Organization Quality of Life-BREF (WHOQOL-BREF) scores for general quality of life, health satisfaction, four domains, and eight facets, with significant QoL differences among three standard localized prostate cancer treatments. A 95% confidence interval is illustrated as a colored shadow for each specific item score. ^a^ Score of facets in the WHOQOL-BREF. Patients under observation were also included in the active surveillance group. AS, active surveillance; RP, radical prostatectomy; RT, radiotherapy; QoL, quality of life; WHOQOL-BREF, short form of World Health Organization Quality of Life Questionnaire.

Figure 1 shows the dynamic fluctuations in the QoL scores in terms of general QoL, health satisfaction, and four domains among the various treatments for prostate cancer in the time after diagnosis. Patients receiving RT appeared to have lower mean scores in the physical domain at the beginning of treatment, which gradually disappeared at around 30 months later. Higher health satisfaction scores were noted in patients receiving RT compared with those receiving RP 40 months after treatment. However, any inference should be drawn with caution because of the relatively fewer patients enrolled or the small sample size after this time frame. The QoL scores were similar among the three groups in terms of general QoL and in the psychological, social, and environmental domains. Dynamic changes in the scores for eight facets with significant QoL differences among treatments are shown in Figure 1. Patients receiving RT had higher QoL pain scores at around 8 to 30 months after treatment, yet they had lower scores in terms of work capacity, transportation, and daily information compared with patients receiving RP or AS in the first 2–3 years after treatment. In contrast, patients under AS treatment had consistently lower scores for negative feelings, and the RT group caught up with their high scores related to sleep and respect by 20 months after treatment.

### 3.2. Determinants of QoL

Table 3 summarizes the potential effects of the various determinants and comorbidities. We found that family income had positive impacts on the scores of all four domains of the WHOQOL-BREF and on many of the facets. As anticipated, we found that patients with diabetes had lower scores in the overall health, physical, and psychological domains and in many related facets, while those with anxiety/depression were affected in the scores of the physical domain and in facets of dependence on medical, sleep/rest, self-esteem, and negative feeling. Patients with an intermediate risk group showed statistically significant higher scores in the social domain and related facets, including sexual activity, compared with those with a high risk.

After controlling for the major clinical and sociodemographic factors, Table 4 shows that patients with AS had lower scores in the psychological domain and related facets before treatment, but higher scores in the physical and social domains and facets of sleep and rest, and personal relationship in the first year compared with the RP group. The RT group had a poorer work capacity in the first year compared with the RP or AS groups, and poorer social domain scores, social support, and being respected compared with the AS group. No significant differences in QoL were found beyond one year after treatments.

## 4. Discussion

The dynamic changes in generic QoL in PC patients among the various treatments were demonstrated in this study using a kernel smoothing method and a mixed effect analysis. Factors including financial income, diabetes, anxiety or depression, risk group, and urology medication usage showed significant effects on QoL. These results indicated that not only QoL in the specific aspects related to adverse effects resulting from individual treatment, but also generic QoL, are significantly impacted by various PC management practices.

Although this was a single institute analysis, the results that could be extrapolated for our participants shared similar clinical features with patients registered in the TCR, with the exception of the older patients and a lower arthritis comorbidity in the TCR. As there were only five diagnoses in the hospitalization records and three diagnoses in the outpatient visits acquired from the NHI data, the extremely low incidence of arthritis in patients from the TCR may be as a result of the low priority of this diagnosis for reimbursement, possibly causing an underestimation.

It is evident that QoL is significantly influenced by the PC management option [19]. However, studies on the issue of QoL, such as Prostate Testing for Cancer Treatment (PROTEC T), mainly revealed differences in cancer specific QoL instead of the generic QoL [20]. This can be attributed to the potential misclassification in the PROTEC T trial, where up to 33.6% of the patients in the AS group changed to RP or RT within 5 years. The cross reaction due to different treatments would also be a potential concern in the same patient receiving subsequent management, such as patients receiving RP followed by adjuvant RT, which might complicate the effects of each treatment in terms of generic QoL. We successfully demonstrated the dynamic differences in generic QoL in patients receiving unitary treatment modalities (Figure 1) and revealed the differences in the physical and social domains with five QoL items after controlling for the major confounding factors (Table 3).

The QoL trajectories of RT patients indicated poorer physical domain, work capacity, and environmental resources from the beginning of treatment, which illustrates the tendency in clinical selection. The phenomena correlate with the relatively poor clinical and sociodemographic picture of patients receiving RT in other studies [21]. Poor mobility of RT in the early phase after treatment may also be considered as the result of poor physical function combined with bowel dysfunction. Interestingly, scores in the facet of pain or discomfort appeared to be better at around 10 months, up to 30 months after RT treatment, compared with the other groups. These findings were not significant after controlling for potential confounders in the subsequent mixed model. Patients under AS reported good physical domain scores and good feelings of respect in the first 2 years in our study. This result is compatible with the current consensus that AS has the least negative impact on QoL compared with RP or RT [22].

Clinical and sociodemographic factors also play important roles as determinants of QoL in localized PC survivors [11]. Household income had extensive positive effects on all domain scores and on 15 facet scores. This result corroborated the majority of current studies that income has a positive association with health related QoL to some degree [23]. Decreased overall health, lower scores in the domains of physical, psychology, and poor eating of desired food in patients with diabetes were also compatible with the clinical practice and the findings of a previous study [24]. Patients reporting anxiety or depression exhibited poor overall health, poor physical domain scores, medical dependence, poor sleep and rest, and poor self-esteem and negative feelings, which matched the results of previous studies, indicating that people with low-level depressive symptoms, as measured by Geriatric Depression Scale, experience significant effects on QoL in the physical and psychological domains of the WHOQOL-BREF [25]. These findings increased the validity of our study. After controlling for confounders, the superior QoL in patients under AS within the first year after disease management remained. The differences in QoL among the various PC treatments diminished after one year. This indicated that dynamic changes in QoL will gradually correct themselves over time, as is the case with functional outcomes [9,26].

There were some limitations in this study. The standard PC managements enrolled were not further sub-grouped, such as stratified radical prostatectomy into open, laparoscopic, or robotic assisted techniques, because there were not enough subjects to generate a reliable comparison. Nonetheless, the impact may have been minimal, as the functional and QoL outcomes appeared to be similar among these techniques [27]. Second, we used the WHOQOL-BREF instead of the typical generic QoL assessment tool used in PC survivors, such as the Short Form Health Survey (SF-36) or the Functional Assessment of Cancer Therapy (FACT-P). However, WHOQOL-BREF has demonstrated validity and reliability in general populations and is being increasingly employed in PC patients [13,28]. It might serve as an ideal tool for measuring generic QoL with additional benefits of cross-cultural validity. Finally, despite the fact that our participants shared similar clinical characteristics with the PC patients in the TCR, our study was still non-randomized in design, with a relatively small number of patients, especially in the RT group. Further large-scale prospective studies are needed to confirm these results.

## 5. Conclusions

There are dynamic differences in generic QoL among localized PC patients receiving different primary treatments. The major effects of different treatments on QoL lies in the first year after treatment. Household income and comorbidities including diabetes and anxiety also have profound impacts on QoL. The results provide additional information for shared decision-making in the treatment of localized PC.

## Figures and Tables

**Table 1:** Baseline demographic and clinical characteristics among participants stratified by treatment.

Disease Status	Radical Prostatectomy	Radiotherapy	Active Surveillance	*p*
Number of subjects (*n*)	104	37	55	
Number of assessments (*n*)	288	96	107	
Age at diagnosis, mean (SD) years	65.3 (5.66)	71.7 (4.96)	71.4 (8.02)	0.001
Education, years, mean (SD) years	12.4 (4.51)	10.8 (4.32)	10.5 (5.10)	0.02
Incomes (per 10^4^ NTD per month)	5.74 (5.95)	3.09 (2.99)	4.90 (5.75)	0.04
Employment, *n* (%)				
Employed	50 (48)	10 (27)	16 (29)	0.02
Unemployed	54 (51)	27 (73)	39 (71)	
Comorbidities, *n* (%)				
Cardiovascular disease	18 (17)	8 (21)	8 (14)	0.68
Hypertension	35 (33)	12 (32)	21 (38)	0.81
Diabetes	26 (25)	6 (16)	8 (14)	0.23
Arthritis	8 (8)	7 (18)	7 (12)	0.16
Anxiety ^†^ (first assessment)	15 (14)	6 (16)	4 (7)	0.34
Anxiety ^†^ (later assessment)	32 (17)	9 (15)	8 (15)	0.89
Peptic ulcer	3 (3)	2 (5)	4 (7)	0.44
Risk group, *n* (%)				
Low	19 (18)	5 (13)	19 (34)	0.02
Intermediate	47 (45)	8 (22)	17 (31)	
High	38 (37)	24 (65)	19 (34)	
Prostate size at diagnosis, mean (SD) mL	39.2 (19.2)	44.4 (24.9)	43.1 (24.0)	0.36
ADL score (1st assessment), mean (SD)	66.6 (39.1)	67.7 (39.8)	79.5 (34.8)	0.11
Domain score (1st assessment), mean (SD)				
Physical	14.4 (2.02)	14.1 (2.26)	14.4 (2.43)	0.72
Psychology	12.8 (1.85)	12.5 (1.52)	12.6 (1.82)	0.66
Social	13.7 (2.30)	13.7 (2.85)	14.0 (2.60)	0.81
Environment	14.4 (1.95)	14.1 (2.26)	14.2 (2.19)	0.73

^†^ Anxiety included depression; ADL–Barthel Index for activities of daily living. Patients under observation were also included the active surveillance group.

**Table 2:** Baseline clinical characteristics of participants at the National Cheng Kung University Hospital (NCKUH) and patients with localized prostate cancer registered in the Taiwan Cancer Registry (TCR).

	NCKUH (*n* = 196)	TCR (*n* = 31,183)	*p*
Age at diagnosis, mean (SD) years	68.24 (7.00)	72.82 (9.16)	<0.01
Comorbidities, *n* (%)			
Cardiovascular disease	34 (17)	6667 (21)	0.17
Hypertension	89 (45)	15,968 (51)	0.10
Diabetes	40 (20)	5610 (18)	0.38
Arthritis	22 (11)	48 (0.1)	<0.01
Anxiety/depression	18 (9)	2742 (9)	0.84
Peptic ulcer	23 (12)	4215 (14)	0.46
Risk group ^a^, *n* (%) ^b^			
Low	43 (22)	3983 (25)	0.62
Intermediate	72 (37)	5643 (35)	
High	81 (41)	6321 (40)	
Primary prostate treatment, *n* (%) ^b^			
Radical prostatectomy	104 (53)	12,942 (46)	0.11
Radiotherapy	37 (19)	5311 (19)	
Active surveillance	55 (28)	9670 (35)	

Patients were diagnosed with prostate cancer in NCKUH from 2009 to 2017 and in TCR from 2009 to 2016. ^a^ Prostate cancer risk group information in TCR was limited to 2012–2016 because of availability. ^b^ Patients with incomplete information on risk group or primary prostate cancer treatment were not analyzed in the statistics. SD, standard deviation.

**Table 3:** Regression coefficients and standard errors (in parentheses) of fixed effects (or major determinants) for mixed effects models of domains and facet scores of the WHOQOL-BREF.

	Fixed Effects	Income (per 10^4^ NTD)	DM (Yes/No)	Arthritis (Yes/No)	Anxiety ^a^ (Yes/No)	Risk Group ^b^ (Int. vs. High)	Uro. Med. (yes/no)
QoL Score							
Overall QoL					−0.43 (0.12) *		
Overall health			−0.30 (0.13) *				
Physical domain		0.07 (0.03) ^†^	−0.88 (0.36) *		−1.11 (0.39) *		−0.46 (0.21) *
Pain and discomfort							−0.20 (0.09) *
Depend on medical		0.02 (0.01) *	−0.56 (0.14) ^‡^		−0.53 (0.17) *		
Energy and fatigue		0.03 (0.01) *					
Mobility			−0.35 (0.14) *	−0.42 (0.18) *			
Sleep and rest					−0.62 (0.15) *		
Daily activities		0.02 (0.01) *					
Work capacity		0.02 (0.01) *	−0.31 (0.11) ^†^	−0.31 (0.14) *			−0.19 (0.09) *
Psychological domain		0.07 (0.02) ^†^	−0.66 (0.27) *				
Spirituality		0.03 (0.01) ^†^	−0.34 (0.12) ^†^				
Bodily image		0.02 (0.01) ^†^					
Self-esteem		0.03 (0.01) ^†^	−0.28 (0.11) *		−0.38 (0.13) *		
Negative feelings					−0.58 (0.17) *		
Social domain		0.09 (0.03) ^†^				1.13 (0.39) ^†^	
Personal rel.		0.02 (0.01) ^†^				0.24 (0.11) *	
Sexual activity						0.42 (0.19) *	
Social support		0.02 (0.01) ^†^				0.24 (0.10) *	
Environment domain		0.10 (0.03) ^‡^					
Eating/food		0.02 (0.01) *	−0.34 (0.11) ^†^				
Home		0.03 (0.01) ^‡^					
Financial		0.05 (0.01) ^‡^					
Information		0.03 (0.01) ^†^					
Physical env.		0.03 (0.01) ^†^					
Transportation		0.03 (0.01) *					

All models are adjusted for age, education, employment (yes/no), cardiovascular disease (yes/no), hypertension (yes/no), peptic ulcer (yes/no), primary prostate treatment (radical prostatectomy/radiotherapy/active surveillance or watchful waiting), post-treatment duration (≤1 year vs. before treatment and >1 year vs. before treatment). Anxiety ^a^ included depression. Effects of low vs. high risk group ^b^ are not shown in the table because of no significant difference in the QoL items for these fixed effects. * *p* < 0.05, ^†^ *p* < 0.01, ^‡^ *p* < 0.001; those with *p* ≥ 0.05 are left blank. WHOQOL-BREF, short form of the World Health Organization Quality of Life Questionnaire; NTD, New Taiwan dollar; QoL, quality of life; DM, diabetes mellitus; Uro. Med., urology medication; Personal rel., personal relationships; Env., environment.

**Table 4:** Regression coefficients in the interaction between prostate cancer treatment and post-treatment duration with standard errors (in parentheses) on a mix-effects WHOQOL-BREF model.

Treatment		Radiotherapy vs. Radical Prostatectomy			Active Surveillance vs. Radical Prostatectomy			Radiotherapy vs. Active Surveillance		
	Time ^b^	before Treatment	after Treatment ≤ 1 Year	after Treatment > 1 Year	before Treatment	after Treatment ≤ 1 Year	after Treatment > 1 Year	before Treatment	after Treatment ≤ 1 Year	after Treatment > 1 Year
QoL Items ^a^										
Physical domain						1.11 (0.53) *			−1.53 (0.64) *	
Sleep and rest						0.63 (0.21) ^†^				
Work capacity			−0.44 (0.20) *						−0.75 (0.24) ^†^	
Psychological domain					−0.93 (0.42) *					
Spirituality					−0.40 (0.20) *					
Self-esteem					−0.34 (0.17) *					
Social domain						1.62 (0.66) *			−1.74 (0.80) *	
Personal rel.						0.34 (0.17) *				
Social support									−0.49 (0.19) ^†^	
Respect									−0.53 (0.23) *	

Mixed effects model adjusted for age, education, income, employment (yes/no), cardiovascular disease (yes/no), hypertension (yes/no), diabetes (yes/no), arthritis (yes/no), anxiety/depression (yes/no), peptic ulcer (yes/no), risk group, and urology medication (yes/no). QoL items ^a^ include the domain and facets from the WHOQOL-BREF, which had major effects on the determinants. time ^b^ post-treatment duration. Patients under watchful waiting were also included the active surveillance group. * *p* < 0.05, ^†^ *p* < 0.01; those with *p* ≥ 0.05 are left blank. Patients under observation were also included in the active surveillance group. WHOQOL-BREF, short form of the World Health Organization Quality of Life Questionnaire; ref, reference; QoL, quality of life; Personal rel., personal relationships.

## Data Availability

The data presented in this study are available on request from the corresponding author.
